# Late‐onset bipolar disorder with dementia: A review of Japanese case reports

**DOI:** 10.1002/pcn5.117

**Published:** 2023-06-30

**Authors:** Yasuhito Nagai, Narihiro Orimo, Shuntaro Natsume, Takumi Hirose, Takao Saida, Eiji Kirino

**Affiliations:** ^1^ Department of Psychiatry Juntendo University School of Medicine Bunkyoku Tokyo Japan; ^2^ Department of Psychiatry Juntendo University Shizuoka Hospital Nagaoka Izunokunishi Japan; ^3^ Department of Psychiatry Juntendo Tokyo Koto Geriatric Medical Center Kotoku Tokyo Japan

Late‐onset bipolar disorder (LOBD) and mania are not well understood and have not been thoroughly investigated. Old‐age bipolar disorder (OABD) is classified into LOBD and early‐onset bipolar disorder (EOBD). LOBD is characterized by a manic/hypomanic episode first occurring in old age in a patient with no known clinical history of the disorder, whereas EOBD represents continuation of the illness at older ages in patients with a long‐standing clinical history of bipolar disorder.^1^, ^2^ Consensus is lacking regarding an age cutoff for LOBD versus EOBD. A common metric is 60 years of age, although the International Society for Bipolar Disorder has proposed a cutoff of 50 years and some groups have proposed 40 years.^2^ Late‐onset mania has a broad differential diagnosis because mania can arise as part of OABD in patients with a history of recurrent depression, schizoaffective disorder, secondary mania (with a specific medical cause), and accompanying delirium and dementia. Angst et al.^3^ reported a 1% annual rate of diagnostic change from unipolar to bipolar I disorder and 0.5% for bipolar II disorder. Population‐based surveys revealed a decrease in the prevalence of bipolar disorder with age, but 6%–8% of all new cases of bipolar disorder develop in persons ≥60 years of age.^4^, ^5^ In recent years, history of bipolar disorder has been thought to be a risk factor for dementia in old adults.^6^ Considering population growth and aging, the prevalence of LOBD and late‐onset mania with dementia is expected to increase, and these conditions will be more common. Sami et al.^7^ reviewed 35 published cases of late‐onset mania and found that 82% had a postulated organic cause, including vascular or drug‐induced etiology and electrolyte imbalance. However, because of language barriers, many Japanese case reports were not included. Thus, we aimed to review published Japanese case reports to deepen our understanding of LOBD in the Japanese population.

We performed a literature search in ICHUSHI (https://www.jamas.or.jp/), a popular search engine for Japanese medical literature, using Japanese words equivalent to the following terms: (bipolar OR mania) AND (elderly OR old age OR late‐onset). Studies were limited to the Japanese language and human studies without data restriction. Databases were searched through March 2022. The inclusion criteria were published and peer‐reviewed case reports that contained detailed case presentations of patients with a manic or hypomanic episode presenting over the age of 50 years. Exclusion criteria were cases in which the onset of mania or hypomania was unclear or in which the age at the first episode was not specified. Cases in which the diagnosis did not use international diagnostic criteria (International Statistical Classification of Disease and Related Health Problems‐10 or Diagnostic and Statistical Manual of Mental Disorders) were also excluded. We identified 25 cases and excluded 12 according to the criteria. Of 13 cases (Table 1), 11 were LOBD, one was EOBD, and one was secondary mania. The implicated cause was antidepressant use in four cases (36%) and a vascular factor in three cases (27%). A total of seven cases (64%) were comorbid with dementia. Of note, vascular factor in Table 1 was a possible factor for manic episode; the authors did not conclude that the manic episode was caused by this factor in the case reports. In a similar study, Sami et al. concluded that dementia was present in 9% of those with late‐onset mania. Compared to these results, it appears that dementia is more common in Japanese case reports for individuals with LOBD. Our review included a smaller number of cases compared to the earlier study, likely due to more restriction in the criteria used by our design. The difference in dementia rates may be due to this restriction. In Japan, the population is aging more rapidly and antecedently growing, which may also affect the result. According to a case‐register study,^8^ among psychiatry outpatients, those with bipolar disorder had the highest rate of dementia onset. This result is in line with the findings of a recent meta‐analysis of 10 studies showing a threefold increased risk of dementia among those with bipolar disorder, with a higher odds ratio than major mood disorder.^9^ Thus, with an increasing number of elderly patients with bipolar disorder, the possibility of dementia is greater. This review contained a small number of cases and had a publication bias. Although a case study is of limited generalizability, we can conclude that LOBD with dementia is common.

**Table 1:** List of old age bipolar disorder in Japanese case reports.

Case (number of times a reference is repeated)	Age of onset	Psychosis	Criteria	Imaging tests	Implicated causative factor	Psychiatric history	MMSE HDS‐R	BD type	Dementia type
Kitamura et al. (2011) (1)	89		DSM‐4TR	MRI: left hippocampus mild atrophy			20/19	LOBD	AD
				SPECT: decreased blood flow in left temporal and parietal lobe					
Kitamura et al. (2011) (2)	81	Grandiose and persecutory delusions	DSM‐4TR	CT: mild diffuse cortical atrophy			22/24	LOBD	UND
Kitamura et al. (2011) (3)	76		DSM‐4TR	CT: mild diffuse cortical atrophy				LOBD	UND
Watanabe (2017) (1)	70s	Persecutory delusions	ICD‐10	MRI: diffuse cortical atrophy and hippocampus moderate atrophy			10/13	LOBD	AD
Watanabe (2017) (2)	80s	Persecutory delusions	ICD‐10	MRI: hippocampus atrophy and parietal cortical atrophy	Donepezil		16/13	LOBD	AD
				SPECT: decreased blood flow in precuneus, posterior cingulate gyrus and parietal lobe					
Watanabe (2017) (3)	70s	Auditory and visual hallucinations, cenesthopathy	ICD‐10	MRI: left > right hippocampus atrophy, old lacunar infarction in left frontal cortex	Left frontal subcortical infarcts, donepezil	Depression	22/24	LOBD	DLB
				DAT scan: decreased dopamine uptake in striatum					
Watanabe (2017) (4)	80s	Auditory and visual hallucinations, delusions of infidelity	ICD‐10	MRI: mild diffuse cortical atrophy	Sertraline		20/21	LOBD	DLB
				DAT scan: decreased dopamine uptake in striatum					
Hosomi (2007) (1)	72	Poverty delusion	DSM‐3‐R	No				EOBD	×
Hosomi (2007) (2)	72	Poverty and persecutory delusion	DSM‐3‐R	No	Clomipramine	Depression		LOBD	×
Sanada et al. (2006)	70	Persecutory delusions	DSM‐4	MRI: infarction in right basal ganglia to radiate crown	Cerebral infarcts		NA/24	Secondary mania	×
				SPECT: decreased blood flow in right temporal lobe and thalamus					
Amino et al. (2010)	67		DSM‐4TR	CT: low‐density area in frontal lobe	Leukoariosis, fluvoxamine			LOBD	×
Mukai et al. (2008)	65		DSM‐4TR	CT: within normal limit	Amitriptyline	Depression		LOBD	×
Koganemaru et al. (2011)	74		DSM‐4TR	MRI: moderate diffuse cortical atrophy, T2 high intensity in cerebral deep white matter	Chronic ischemic change, paroxetine	Depression	28/29	LOBD	×

Abbreviations: AD, Alzheimer disease; BD, bipolar disorder; CT, computed tomography; DAT scan, dopamine transporter single–photon emission computed tomography; DSM, diagnostic and statistical manual of mental disorders; DLB, dementia with Lewy bodies; EOBD, early‐onset bipolar disorder; HDS‐R, hasegawa dementia scale–revised; ICD‐10, international statistical classification of disease and related health problems–10; LOBD, late‐onset bipolar disorder; MMSE, mini–mental state examination; MRI, magnetic resonance imaging; NA, not applicable; R, revised edition; SPECT, single–photon emission computed tomography; TR, text revision; UND, unspecified neurocognitive disorder.

## AUTHOR CONTRIBUTIONS

Yasuhito Nagai conceived the idea of the study. Yasuhito Nagai and Narihiro Orimo were involved in data interpretation. All authors participated in discussion, writing the manuscript, and revisions. All authors read and approved the final version of the manuscript.

## CONFLICT OF INTEREST STATEMENT

The authors declare no conflicts of interest.

## ETHICS APPROVAL STATEMENTS

N/A.

## PATIENT CONSENT STATEMENT

N/A.

## CLINICAL TRIAL REGISTRATION

N/A.

## Supporting information

Supporting information.

## Data Availability

N/A.
