# Supplementary material for: Late‐onset bipolar disorder with dementia: A review of Japanese case reports
Source: PCN Rep. 2023 Jun 30;2(3):e117. doi: 10.1002/pcn5.117 (PMC11114427; doi:10.1002/pcn5.117)
Supplement: Supplementary file 1 — Supporting information. [file PCN5-2-e117-s001.docx]

**Supporting information** (Reference of the table)

Amino K, Osawa Y, Katayama S, Iimori M Successful use of cilostazaol in treatment of late-onset bipolarⅡdisorder :A case report Clinical psychiatry 2010 52:1205-1208 (in Japanese)

Hosomi M Old age delusion, 2 cases of senile depression in patients with bipolar disorder. Journal of Jinmeikai Research Institute of mental health 2007 4(1) 62-67 (in Japanese)

Kitamura T, Tanaka N, Kitamura M, Shibuya R, Kurata K, 3 cases of dementia with elderly-onset mania. Japanese journal of geriatric psychiatry. 2011 22: 203-210 (in Japanese)

Koganemaru G, Nagatomo K, Ishida Y A case of late-onset bipolarⅠdisorder with deep white matter lesions. Japanese journal of clinical psychiatry 2011 40: 1365-1370 (in Japanese)

Mukai Y A case of late-onset bipolar depression-psychomotor retardation and melancholic depression (Paker G et al) Japanese journal of clinical psychiatry 2008 37: 1257-1265 (in Japanese)

Sanada K, Owashi T, Shishikura K, Tukahara K, Takashio O, Otsubo T, Mimura M, Kamijima K A patient with mania of senile onset. Clinical psychiatry 2006 48:1029-1031 (in Japanese)

Watanabe K. late-onset bipolar disorder after dementia progression along with first onset of mania. Japanese journal of clinical psychiatry.2017 46:1353-1361 (in Japanese)
